# Nursing green transformational leadership style, behavioral intentions, actual behavior and creativity: the impact of a green climate

**DOI:** 10.1186/s12912-025-03331-2

**Published:** 2025-06-17

**Authors:** Nagah Abd El-Fattah Mohamed Aly, Safaa M. El-Shanawany, Maha Abdelhamied Ghanem, Wael M. Lotfy

**Affiliations:** 1 Nursing Administration, Faculty of Nursing, Matrouh University, Marsa Matrouh, Egypt; 2 Forensic Medicine and Clinical Toxicology, Faculty of Medicine, Alexandria University, Alexandria, Egypt; 3 Community Health Nursing, Faculty of Nursing, Matrouh University, Marsa Matrouh, Egypt

**Keywords:** Green transformational leadership, Environmental behavior intention, Actual green behavior, Green creativity, Green work climate, Mediating effect, Moderating effect, Nurses, Nursing management

## Abstract

**Background:**

Nursing leaders and nurses play a significant role in promoting green healthcare practices in healthcare organizations. Therefore, it is essential to understand the mechanism through which leadership can affect the green behavior and creativity of nurses.

**Aims:**

To investigate the effects of transformational leadership and a green work climate on nurses’ behavioral intentions, actual green behavior, and green creativity.

**Methods:**

Self-report questionnaires (nursing green transformational leadership, climate, behavior, and creativity) were used to gather data from 400 nurses.

**Results:**

Nurses’ perceptions of nursing green transformational leadership, climate, behavior, and creativity were all at acceptable levels. Regression analysis indicated that nurses’ green behavioral intentions, actual green behavior, and green creativity, as well as the green work climate, were positively affected by nursing green transformational leadership. The Sobel results showed that nursing green transformational leadership indirectly affected nurses’ green behavioral intentions, actual green behavior and green creativity through the mediating role of the green work environment. There was a moderating influence of the green work climate on the association between nurses’ green behavioral intentions and actual green behavior.

**Conclusion:**

A green work climate had a mediating effect on the relationships among nursing green transformational leadership, nurses’ green behavioral intentions, actual green behavior and green creativity. Nurses’ green behavioral intentions positively affect their actual green behavior through the moderating effects of a green work climate.

**Implication:**

Nurse managers should be aware of the influence of leadership style and the green work climate as significant factors influencing the ability of nurses to engage in green behavior and foster their green creativity.

**Clinical trial number:**

Not applicable.

## Introduction

Green management concepts and practices have become a growing area of interest among businesses, academics, and healthcare institutions worldwide to achieve sustainable development goals and mitigate the adverse effects of environmental risks and hazards [1]. Green practices have been integrated into various organizational aspects, from green leadership to green processes and practices [2]. 

The green transformational leadership style is an extension and application of transformational leadership theory in the field of environmental responsibility. It focuses mainly on encouraging and supporting proactive environmental protection measures to motivate individuals and organizations to produce environmental behaviors beyond expectations and achieve ecological goals collectively [3, 4]. It influences employees’ voluntary pro-environmental behaviors through internal motivations and emotional states [5]. 

The nursing green transformational leadership style is defined as “behaviors of nurse managers that encourage nurses to perform above expected levels of environmental performance and inspire nurses to attain environmental goals [6, 7]. It plays a critical role in promoting nurses’ innovation, green mindfulness, self-efficacy, intrinsic motivation, behavior, and creativity. It also helps shape the organizational climate [8, 9]. 

A nursing green transformational leader impacts the improvement of an organization’s environmental performance and constructs environmentally friendly values at the individual level when interacting with a green work climate [10–12]. The nursing green work climate can be defined as nurses’ perceptions and interpretations of organizational policies, procedures, and practices related to environmental sustainability [13]. This climate is a critical contextual factor that shapes nurses’ attitudes, behaviors, and creativity, as well as the translation of green behavioral intentions into actual behavior [13–15]. 

Green behavioral intentions involve employees’ self-set goals to act in an eco-friendly environment at work [13]. The achievement of actual green behavior (GB) among nurses depends primarily on their intentions toward actual GB [16, 17]. Actual GB is an individual activity that minimizes harm to or benefits the natural environment (i.e., general GB) [18]. GB is defined as “scalable actions and behaviors that employees engage in, are linked with, and contribute to or detract from environmental sustainability” [19]. 

In this respect, the componential theory of creativity suggests that individuals’ creativity can be influenced by the social or work climate. The work climate can stimulate the creative behavior of employees, e.g., nurses, when subordinates are motivated and possess relevant skills [15]. Therefore, a green work climate can motivate them to engage in more innovative activities to foster green creativity. Nurses’ green creativity is referred to as their potential and role in generating and initiating green innovative ideas [20]. 

Recently, many studies have focused on explaining the influence of servant and ethical leadership styles on the engagement of nursing staff in pro-environmental behavior and creativity in the workplace [16, 21–23]. To our knowledge, in Egypt, one study used a training program to explore the relationship between a green transformational leadership style in nursing and nurse managers’ behavior and creativity [24]. However, the mechanisms by which green transformational leadership influences nurses’ green behavioral intentions, actual GB, and green creativity have not been well studied.

Considering the role of a green work climate as a mediator and moderator in the healthcare system can help in understanding the mechanisms by which green transformational leadership influences nurses’ green behavioral intentions, actual GB, and green creativity. This study aims to address this gap.

### Aim of the study

The current study aimed to investigate the effects of transformational leadership and a green work climate on nurses’ behavioral intentions, actual GB, and green creativity through the following:

1. Investigating the relationships among nursing green transformational leadership, the green workplace climate, nurses’ green behavioral intentions, actual GB, and green creativity.
2. Exploring the green climate as a mediating mechanism by which nursing green transformational leadership impacts nurses’ green behavioral intentions, actual GB, and green creativity.
3. Exploring the green climate as a moderating mechanism by which nurses’ green behavioral intentions affect nurses’ actual GB.

### Theoretical approach and hypotheses of the study

**Fig. 1:**
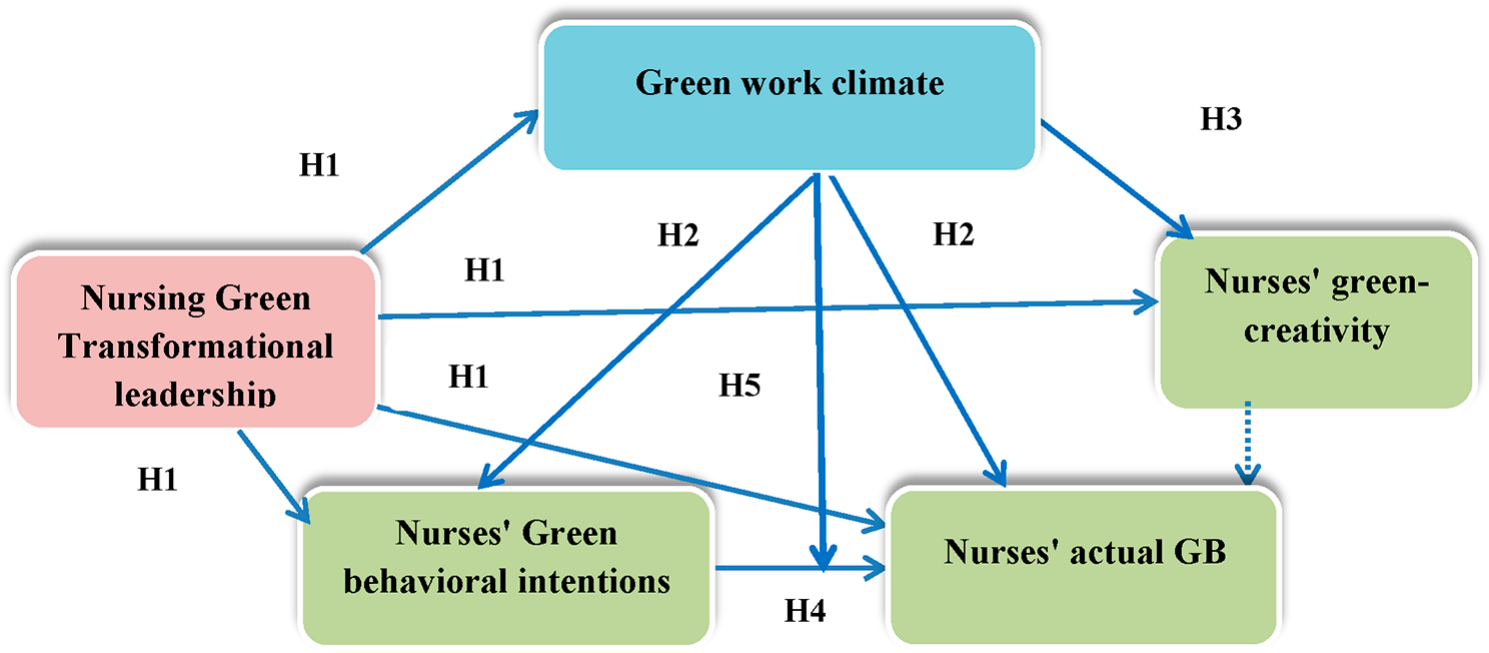
Conceptual diagram of the present study

Nurses and nursing managers, as industrial employees and managers, can take steps toward green activities and practices in their daily work [24]. Applying leadership, behavior and creativity theories is similar in the healthcare, manufacturing, and tourism fields, particularly in the context of environmental sustainability. The healthcare sector can benefit from this approach for prioritizing resource efficiency and minimizing waste, ultimately leading to greener practices. By fostering a culture of green creativity and adapting green behavioral strategies, healthcare organizations can create solutions that enhance their operational effectiveness and contribute to a healthier environment. Therefore, the theoretical approach and hypotheses of the present study are as follows (Fig. 1):

### Relationships between green transformational leadership, the green work climate, green behavioral intentions, actual GB, and green creativity

Embedding transformational leadership practices into environmental sustainability gives rise to the concept of “green transformational leadership” [1]. Green transformational leadership can play a definite role in creating a healthier and more sustainable workplace by prioritizing sustainability, promoting actual GB, and establishing a supportive work environment [1, 25]. 

Green transformational leadership influences organizational performance by affecting employees’ psychological climates. In the context of green transformational leadership, leaders prioritizing environmental sustainability can increase employees’ intrinsic motivation by fostering a work environment that values and supports green initiatives [1]. They can also encourage and motivate employees to collaborate and work together to engage in environmentally friendly behaviors in their workplace [4, 26]. 

Green transformational leadership not only influences employees’ attitudes and behaviors toward environmental issues but also enhances employee-green creativity [27]. Based on supported social learning theory, green transformational leaders do this by being creative, setting an example for others, engaging in environmentally friendly behaviors, and demonstrating their dedication to environmental protection through concrete actions. They also serve as role models that can influence the ecological behavior of subordinates. They stimulate employees’ cognitive thinking to solve environmental problems [28]. 

A visionary leader can set the vision of green development by fostering a culture that values the environment. They inspire employees to develop environmental skills, embrace ecological values, and actively participate in environmental initiatives. This results in the exhibition of actual GB and creativity by employees [29, 30]. 

The green transformational leadership style can be better understood through social exchange theory. Social exchange theory, which is based on the concept of reciprocity, has been used to understand employee attitudes, behaviors, and creativity. Transformational leaders establish high-quality reciprocal relationships with their employees by demonstrating care, trust, and support. Consequently, employees are more likely to engage in environmentally friendly behaviors if they feel valued and supported by the organization. They can effectively motivate employees to demonstrate more green creative ideas when they believe that their organization genuinely cares about their ecological concerns and preferences and that the organization articulates a vision that increases their confidence and expectations [1, 31–33).

Previous research has indicated that the elements of transformational leadership have global universality, playing an important role in shaping a green psychological climate within organizations and promoting employee behavior and creativity [31, 32, 34]. On the basis of previous studies and theories, we propose the following hypothesis.

#### H1

Nursing green transformational leadership is positively associated with a green work climate, nurses’ green behavioral intentions, actual GB, and green creativity.

### The mediating role of a green climate in nurses’ green behavioral intentions and actual GB

A green work climate is an essential contextual factor that describes the attitudes and behaviors of employees. A green work climate shapes employees’ perceptions of organizational policies aimed at enhancing sustainability. It can be defined as an employee’s perception of an organization’s potential to enhance green standards through strategies, policies, and procedures that support the environment [19, 35]. 

Social cognitive theory states that the external environment can directly influence employees’ subjective initiative and then shape their behavior [36, 37]. In this context, a green transformational leader can influence a green work climate by developing a mission and vision of green development that supports environmental protection. This enables employees to become aware of their roles and responsibilities concerning green activities. Thus, green transformational leadership influences actual employees’ GB through its effect on the work green climate [19, 35]. 

Social influence theory also proposes that the attitudes, beliefs, and behaviors of an individual can be influenced by others [38]. Social and work interactions between employees and leaders help employees understand the perceptions, rules, and policies of an organization with respect to environmental sustainability [39, 40]. Transformational leaders can influence employees by creating policies, procedures, and a reward system that supports environmental sustainability. This can positively influence employees’ discretionary environmental behaviors [39]. Previous studies in different organizations highlight the role of the work climate as a mediator in promoting employees’ green behavioral intentions and actual GB [35, 40]. The following hypothesis is proposed:

#### H2

A green work climate will play a mediating role in the relationship between nursing green transformational leadership and nurses’ green behavioral intentions and actual GB.

### The mediating effect of a green climate on green creativity

Transformational leadership plays a vital role in creating a green workplace climate that supports creativity [1]. Leaders practicing green transformational leadership foster a green work environment that provides their employees with the autonomy and resources needed to drive innovation and support creativity. They also create a supportive environment that encourages collaboration, open communication, and the exchange of diverse perspectives. All of these are essential elements for creativity to be accomplished [28]. 

The relationship between green transformational leadership and a green climate for creativity is grounded in the componential theory of creativity and social exchange theory [41, 42]. The componential theory of creativity states that the work climate is a factor that stimulates workers’ creativity. Creativity arises when leaders, such as green transformational leaders, create a work climate that enhances employees’ technical and cognitive skills while increasing their intrinsic motivation [28, 41]. Thus, leaders can significantly increase employee creativity, particularly in the context of environmental sustainability [28, 32, 43]. 

According to social exchange theory, when employees perceive organizational support for green activity, they are more likely to engage in creative thinking [42]. Green transformational leaders can influence employees’ emotional and cognitive processes and creativity by shaping their perceptions of the organizational climate [43, 44]. Numerous studies in the manufacturing industry and tourism industry have revealed that a green climate mediates the relationship between green transformational leadership and creativity. Green transformational leadership can enhance employee creativity by fostering a supportive environment that encourages innovative thinking [15, 20, 45, 46]. On the basis of these theories and other studies, we hypothesize the following:

#### H3

The relationship between nursing green transformational leadership and nurses’ green creativity is mediated by a green work climate.

### Relationship between green behavioral intentions and actual GB

On the basis of the theory of planned behavior, behavioral intentions are contiguous with the actual behaviors of employees. Individuals’ behavior is determined by their intentions [17]. Behavior intentions serve as key predictors of actual behaviors within the framework of sustainable settings. The occurrence of actual GB among employees depends primarily on their intentions toward GB [47, 48]. 

A more positive association may exist between employees’ intentions toward GBs and the desire to adopt GBs if employees have strong intentions and are more environmentally sensitive. They may also react better to adopting green practices that are consistent with their values and beliefs. Therefore, individuals who are conscious of the environment are more likely to participate in ecological actions [47, 48]. 

Green behavioral intentions also allow organizations to anticipate the likelihood of nurses engaging in sustainable practices and to design interventions that effectively motivate and encourage actual GB [16, 48]. However, few studies have reported a positive relationship between green behavioral intentions and actual GB [13, 16, 48]. Therefore, investigating nurses’ green behavioral intentions and actual GB toward green activities are considered important issues in healthcare. We hypothesize:

#### H4

Nurses’ green behavioral intentions are positively associated with their actual GB.

### The moderating effect of a green climate

Work climate is defined as the observed entirety of nonmonetary components that establish the conditions in which workers carry out their duties [49]. Organizations may now consider the work climate as a culture, policy, and practice related to a sustainable environment, where employees work and follow green practices [48]. 

The work climate affects employees’ outcomes, such as engagement in green practices, organizational commitment to green practices, and behavior following these practices [39]. One of the elements influencing employees’ behavior is their workplace. If an organization promotes actual GB, these practices are likely to permeate the work environment and promote a green and eco-friendly environment [49, 50]. 

A positive green work climate provides cues to help employees translate their green behavioral intentions into actual GB [16]. In this context, a green work climate plays a critical role in reinforcing the connection between green behavioral intentions and actual GB, leading to increased participation in green activities and creativity [51, 52]. A few studies have focused on how green behavioral intention affects actual GB through the moderating effect of a green climate [13, 16, 48]. On the basis of the above literature reviews, we hypothesize the following:

#### H5

A green work climate moderates the relationship between nurses’ green behavioral intentions and their actual GB; this relationship is stronger when the green work climate perceptions of nurses are acceptable or strong.

## Materials and methods

### **Study settings**

The study was carried out at six Alexandria University hospitals. These hospitals offer a wide range of free medical services for different Egyptian populations. They have a large capacity for occupied beds and many nursing workers. Nurses in these hospitals face sustainable development challenges resulting from managing medical waste, recycling medical materials, energy and water consumption, environmental hazards, and pollution. Nurses in these hospitals have tried to address environmentally friendly practices for promoting nurses, patients, and organizational outcomes.

#### Study design

This was a cross-sectional and descriptive correlational study.

### Study sampling and participants

On the basis of the nurses’ workforce data obtained from the study hospitals, assuming that 50% of health care providers’ workforce (p) was nurses, with an alpha error (z) of 0.05 and a margin of error (d) of 5%, the minimum sample size was 384 nurses, as estimated using Cochran’s formula (Cochran, 1963) (n = z^2^ p (p)/d^2^)[53]. The actual sample size was 400 nurses from six university hospitals who agreed to participate in the study to reduce single-source bias and improve the generalizability of the study.

### Study measures

1. **The control variables** included nurses’ demographic characteristics, such as age, experience, job title, and workplace.
2. **Nursing green transformational leadership scale**: Six rating point items were created by Chen and Chang (2013) [3] to measure nurses’ perceptions of nursing green transformational leadership behaviors, which are displayed by their immediate leaders or managers in their workplace. The alpha coefficient was 0.75.
3. **Green work climate scale**: Six ranking items were adopted from the studies of Norton et al. (2012) [54] to evaluate nurses’ perceptions of the green climate in their workplace. The alpha coefficient was 0.90.
4. **Nurses’ green behavioral intentions**: Six ranking items were developed by Bissing-Olson et al. [55] to measure self-reported task-related (three items) and proactive nurses’ GB (three items) from their perspective. The alpha coefficient was 0.85.
5. **Nurses’ actual GB scales**: Eight rating scale items were established by Robertson and Barling (2013) [56] to quantify the level of actual GB among nurses in their workplace from their perceptions. The alpha coefficient was 0.86.
6. **Nurses’ green creativity scale**: Six items of the rating scale were developed by Chen and Chang (2013) [3] to assess the level of green creativity among nurses from their perspective. The alpha coefficient was 0.95.

### Scoring profile

The nurses were asked to indicate the extent to which they agreed with the questionnaire statements of nursing green transformational leadership, green work climate, nurses’ green behavioral intentions, and green creativity via a 5-point scale ranging from (1 = strongly disagree, 5 = strongly agree). Additionally, they indicated how often they displayed actual GBs on a 5-point scale (1 = not at all, 4 = frequently if not always). The levels of nurses’ perceptions were classified on a 5-point Likert scale as follows: fair level (≤ 3), acceptable level (3–3.9), and good level (≥ 4).

### Psychometric measures

To ensure face and content validity, seven experts in nursing management reviewed the study questionnaires. The experts were asked to provide their comments and any suggestions for any additions or omissions of items. Interrater reliability was achieved using a content validity index (≥ 80%). According to the experts’ comments, no changes were made, as the questionnaires were found to be clear and understandable. To ensure the consistency of the questionnaire, the English language version of the questionnaire was translated into an Arabic language version by researchers via the back translation technique [57].

Test‒retest reliability was assessed via a pilot study with the test‒retest method two weeks apart. The pilot study aimed to ensure the feasibility, clarity, and applicability of the questionnaires and estimate the time required to complete the tools of data collection for each nurse. Forty nurses (10% of the sample size) completed the same questionnaires two times. The pilot sample was not included in the study sample or statistical analysis. The test-retest reliability was 0.78 for nursing green transformational leadership, 0.86 for the green work climate, 0.80 for green behavioral intentions, 0.80 for actual GB, and 0.89 for green creativity.

### Data collection

The data were collected from the study nurses using six self-report questionnaires. Nonresponse rates and decreased response rates among the study nurses were considered potential sources of bias because of the cross-sectional design of the present study. To increase the response rate among nurses, the questionnaires were written in simple and clear Arabic. Book notes and pens were provided.

The researchers also met the nurses to explain the objectives of the study and how to fill out the questionnaires, clarify any doubts, and answer any queries. The nurses chose a suitable moment to complete the study questionnaires during their working hours, and completing the questionnaires took approximately 20 min. The researchers subsequently collected the completed questionnaires at the end of the nurses’ shifts and reviewed each completed questionnaire for completion.

### Statistical analysis

The data were analyzed via SPSS version 25. Descriptive analysis, including percentage, mean, and standard deviation, was used to describe personal characteristics, dependent variables (nurses’ green behavioral intentions, actual GB, and green creativity), independent variables (nursing green transformational leadership) and a mediating and moderating variable (green work climate). Pearson correlation coefficients were used to explore the relationships between the dependent and independent variables and analyze test‒retest reliability. Mediating and moderating analyses were carried out via hierarchical multiple regression analysis. The Sobel test in the mediation analysis was estimated via a free statistical calculator version 4.0 and employed to confirm the mediating effect.

A 5000 bootstrapping test at 95% bootstrap confidence intervals (BC 95% CI) was employed to investigate the mediation model. The SPSS PROCESS macro v4.3 was used to calculate the moderation effect and conditional effects. To avoid the influence of nurses’ demographic characteristics on relationship results, nurses’ demographic characteristics, such as age, experience, job title, and workplace, were measured and controlled. Additionally, the analyses were performed without including control variables. The results were unchanged when the analyses were performed without including age, experience, job title, and workplace in the analysis. Cronbach’s alpha was used to analyze interconsistency reliability.

## Results

### Descriptive analysis of personal characteristics

Nearly half of the nurses (47.5%) were aged between 30 and 45 years, as did nurses who had less than 15 years (49.3%). A total of 49.3% of the nurses in the study units identified their job titles as staff nurses, followed by technical nurses (27.2%) and professional nurses (23.5%). They were working in hospital wards (48.7%), intensive care units (36.0%), and operating rooms (15.3%) (Table 1).

**Table 1:** Distribution of the study sample according to demographic characteristics (*n* = 400*)*

Nurses	No. (%)	Nurses	No. (%)
**Age (Years)**		**Job title**	
< 30	90 (22.5)	Staff Nurse	197(49.3)
30–45	186(47.5)	Professional nurse	94 (23.5)
≥ 45	124(31)	Technical nurse	109 (27.2)
**Experience (Year)**		**Workplace**	
< 15	197(49.3)	Ward	195(48.7)
15–25	94(23.5)	Intensive care units	144(36.0)
≥ 25	109(27.2)	Operating rooms	61(15.3)

### Mean and correlation analysis

The green work climate had the highest mean (3.73), followed by nursing green transformational leadership (3.65), nurses’ green behavioral intentions (3.60), nurses’ green creativity (3.50), and nurses’ actual GB (3.44). Nursing green transformational leadership was positively correlated with the green work climate (*r* = 0.64), nurses’ green behavioral intentions (*r* = 0.72), actual GB (*r* = 0.66), and green creativity (*r* = 0.65) (Table 2).

A green work climate had significantly positive relationships with nurses’ green behavioral intentions (*r* = 0.66), actual GB (*r* = 0.63), and green creativity (*r* = 0.60). A positive relationship was found between nurses’ green behavioral intentions and actual GB (*r* = 0.78) and green creativity (*r* = 0.74), in addition to between nurses’ actual GB and green creativity (*r* = 0.86). All the correlations of the study variables were significant at 0.01 (Table 2).

**Table 2:** Means, standard deviations, and correlations between the study variables

Variables	Min.-Max	* Mean ± SD	1	2	3	4	5
1- Nursing green transformational leadership	1–5	3.65 ± 0.69	1	0.64**	0.72**	0.66**	0.65**
2- Green work climate	1–5	3.73 ± 0.65			0.66**	0.63**	0.60**
3- Nurses’ green behavioral intentions	1–5	3.60 ± 0.97				0.78**	0.74**
4- Nurses’ actual GB	1–5	3.44 ± 0.50					0.86**
5- Nurses’ green creativity	1–5	3.50 ± 0.56					

*fair level (≤ 3), acceptable level (3–3.9), and good level (≥ 4)

**The *p*-value was considered significant at ≤ 0. 01

### Mediating and moderating analyses

**Fig. 2:**
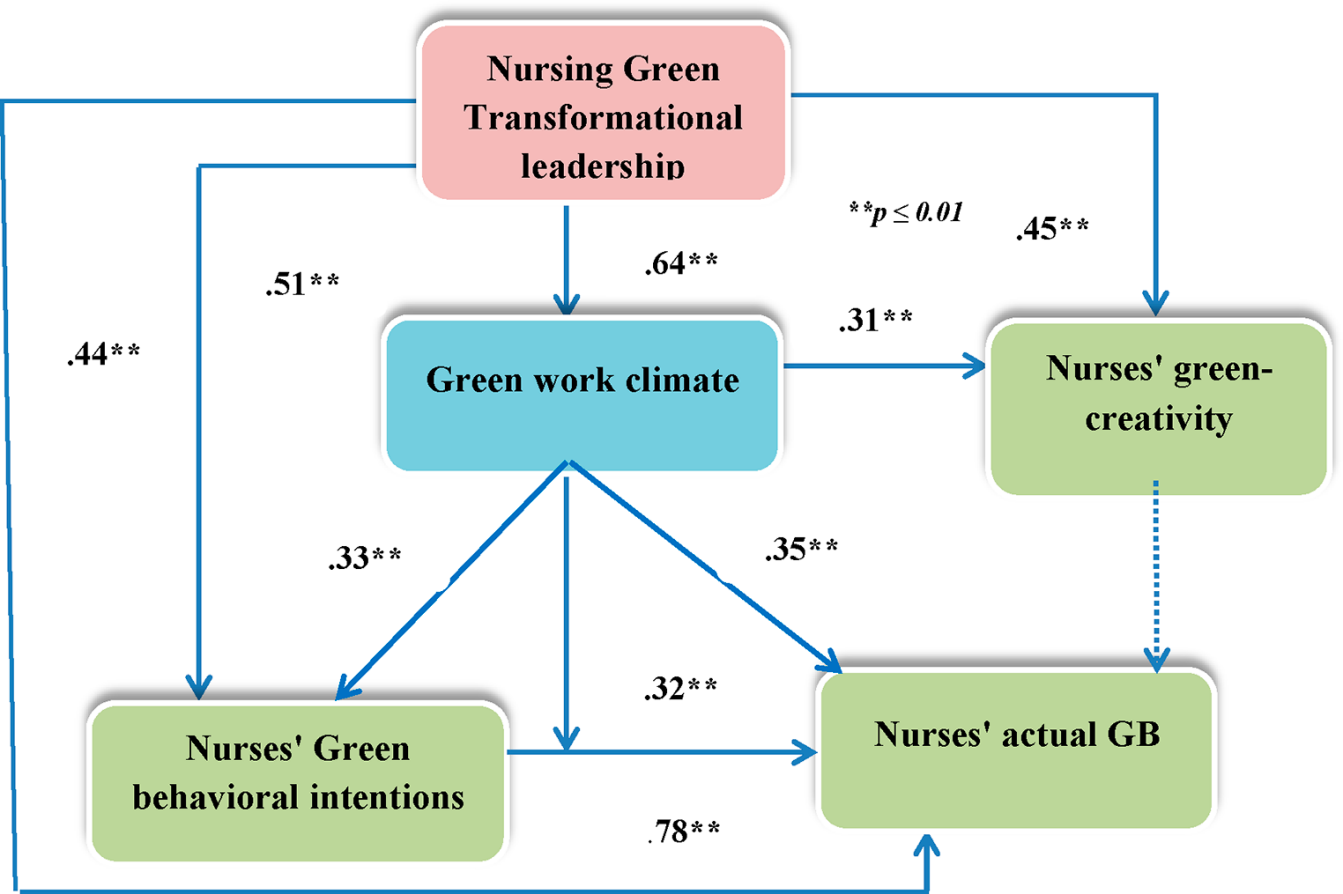
Summaries of the mediating and moderating analyses

The results of the first mediated regression analysis indicated that Models 1 and 2 of nursing green transformational leadership were positively significant (*p* ≤ 0.01) (Table 3; Fig. 2). It explained 41.1% of the green work climate (F = 68.457, *p* = 0.01), 52.2% of nurses’ green behavioral intentions (F = 109.091, *p* ≤ 0.01), 44.7% of actual GB (F = 79.207, *p* ≤ 0.01), and 65.8% of green creativity (F = 75.000, *p* ≤ 0.01). The B value in Model 1 illustrates that if nursing green transformational leadership increases, the green work climate (0.67), nurses’ green behavioral intentions (0.77), actual GB (0.69), and green creativity (0.66) increase (Table 3).

The green work climate as a mediating variable was entered into Model 2 and accounted for approximately 59.2% of the nurses’ green behavioral intentions, 52.1% of the nurses’ actual GB, and 70.0% of the nurses’ green creativity. The green work climate had a positive mediating effect on the relationships among nursing green transformational leadership, nurses’ green behavioral intentions (β = 0.33, *p* ≤ 0.01), nurses’ actual GB (β = 0.35, *p* ≤ 0.01) and nurses’ green creativity (β = 0.31, *p* ≤ 0.01) (Table 3; Fig. 2).

The B value after the addition of the green work climate in Model 2 revealed that when the green work climate increased, the nurses’ green behavioral intentions (B = 0.33), actual GB (B = 0.34), and green creativity (B = 0.29) increased (Table 3).

The results of the Sobel test (z) were found to be significant, indicating the indirect effect of nursing green transformational leadership on nurses’ green behavioral intentions (z = 5.8; p ≤. 01), nurses’ actual GB (z = 5.6; P ≤. 01) and nurses’ green creativity (z = 5.5; P ≤. 01) through green work climate as the mediating variable (Table 3).

**Table 3:** Results of the mediating analysis

Model	Variables	Test (1)			Test (2)			Test (3)			Test (4)		
		Work climate			Behavioral intentions			Actual behavior			Creativity		
		B (SE)	Beta**	t (sig.)	B (SE)	Beta**	t (sig.)	B (SE)	Beta**	t (sig.)	B (SE)	Beta**	t (sig.)
*1*	Leadership	0.67 (0.08)	0.64	8.27 (0.000)	0.77 (0.07)	0.72	10.44 (0.000)	0.69 (0.078)	0.66	8.90 (0.000)	0.66 (0.07)	0.65	8.66 (0.000)
		R square = 0.411 F test = 68.457**			R square = 0.522 F test = 109.091**			R square = 0.447 F test = 79.207**			R square = 0.658 F test = 75.000**		
*2*	Leadership				0.55 (0.09)	0.51	6.05 (0.000)	0.46 (0.09)	0.44	4.82 (0.000)	0.64 (0.09)	0.45	4.86 (0.000)
	**Climate**				0.33 (0.08)	0.33	3.952 (0.000)	0.34 (0.090)	0.35	3.861 (0.000)	0.29 (0.09)	0.31	3.284 (0.000)
					R square = 0.592 F test = 70.488** Sobel Test(z) = 5.8**			R square = 0.521 F test = 52.679** Sobel Test(z) = 5.6**			R square = 0.700 F test = 46.639** Sobel Test(z) = 5.5**		

Unstandardized Coefficients: B and Std. Error (SE); Standardized Coefficients: Beta (β) **p-value was significant at ≤ 0. 01

Model 1 in the moderating analysis, which involved only nurses’ GB intentions, explained 58.1% of the nurses’ actual GB (F = 135.681, *p* ≤ 0.01). Nurses’ green behavioral intentions significantly affected their actual GB (β = 0.76, *p* ≤ 0.01) (Table 4; Fig. 2). The green work climate as a moderator variable was entered into Model 2 and explained 61.2% of the nurses’ actual GB (F = 76.473, *p* ≤ 0.01 0.01). Nurses’ green behavioral intentions (β = 0.60, *p* ≤ 0.01) and green work climate (β = 0.23, *p* ≤ 0.01) had a positive significant influence on nurses’ actual GB (Table 4).

In Model 3, the interaction effect between the green work climate and nurses’ green behavioral intentions was added and explained 84.6% of the nurses’ actual GB (F = 29.337, *p* ≤ 0.01). The R square increased over the models, from 58.1% in Model 1 to 84.6% in Model 3 (Table 4). The interaction effects of green work climate and nurses’ green behavioral intentions on actual GB were found to be statistically significant, indicating that the relationship between nurses’ green behavioral intentions and actual GB was moderated by the green work climate (β = 0.32, *p* ≤ 0.01) (Table 4; Fig. 2). An increasing effect of the green work climate on nurses’ actual GB was found when the climate interacted with nurses’ green behavioral intentions (B = 0.30) (Table 4).

**Table 4:** Results of moderating analysis

Model	Variables	Nurses’ actual GB				
		B (SE)	Beta	t (sig.)	*R* square	F test (sig.)
1	Nurses’ green behavioral intentions	0.74 (0.06)	0.76**	11.648(0.000)	0.581	135.681(0.000)**
2	Nurses’ green behavioral intentions	0.59 (0.15)	0.60**	7.178(0.000)	0.612	76.473 (0.000)**
	Green work climate	0.23 (0.08)	0.23**	2.797(0.006)		
3	Nurses’ green behavioral intentions	0.76 (0.16)	0.78**	4.521(0.000)	0.846	29.337 (0.000)**
	Green work climate	0.37 (0.15)	0.37**	3.699(0.000)		
	Interaction (green behavioral intentions * green work climate)	0.30 (0.12)	0.32**	2.917 (0.004)		

Unstandardized Coefficients: B and Std. Error (SE); Standardized Coefficients: Beta (β) **p- value was significant at ≤ 0. 01

### Bootstrapping analysis of mediating effects

The role of the green work climate as a mediator between nurses’ green behavioral intentions and actual GB was examined by bootstrapping analysis with a 5000 repeated sample size. Significant direct impacts of green transformational leadership on nurses’ green behavioral intentions (β = 0.54), actual GB (β = 0.46), and green creativity (β = 0.46) were found when the green work climate was a mediator (Table 5).

The total effect of a green work climate on nurses’ green behavioral intentions was 0.77, the actual GB was 0.69, and the level of green creativity was 0.66. Indirect impacts of green transformational leadership on nurses’ green behavioral intentions (β = 0.33), actual GB (β = 0.34), and green creativity (β = 0.29) were demonstrated when the green work climate was a mediator. The BC 95% CI did not include 0, indicating the mediating role of the green work climate (Table 5).

**Table 5:** Bootstrapping analysis of mediating effects

Relations	Total effect				Direct				Relations	Indirect effect			
	Β	SE	BC 95% CI		β	SE	BC 95% CI			β	SE	BC 95% CI	
			LL	UL			LL	UL				LL	UL
NGTL→GBI	0.77	0.08	0.596	0.915	0.54	0.12	0.244	0.826	NGTL→GWC→GBI	0.33	0.14	0.252	0.813
NGTL→AGB	0.69	0.09	0.508	0.853	0.46	0.15	0.159	0.766	NGTL→GWC→AGB	0.34	0.15	0.155	0.766
NGTL→GC	0.66	0.08	0.475	0.818	0.46	0.18	0.155	0.759	NGTL→GWC→GC	0.29	0.15	0.245	0.872

NGTL (nursing green transformational leadership); GBI (green behavioral intentions); AGB (actual green behavior); GC (green creativity); GWC (green work climate); *n* = 400; bootstrap sample size = 5000, BC 95% CI = bootstrap confidence intervals

### Conditional test of the moderation effect

**Fig. 3:**
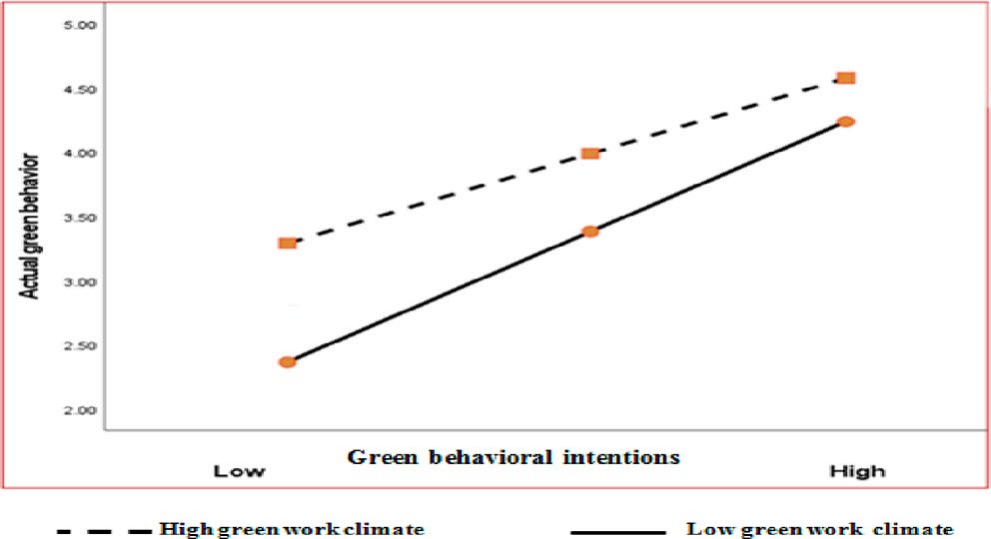
Moderating effect of a green work climate on the relationship between green behavioral intentions and actual GB

The positive relationship between green behavioral intentions and actual GB was moderated by the green work climate. The conditional effect of green behavioral intentions was positively stronger and more significant at high and moderate levels of the green work climate (conditional effect = 0.66 and 0.55; SE = 0.09 and 0.08; *t* = 7.19 and 6.74; 95% confidence intervals of 0.48-0.85 and 0.39-0.71; *p* < 0.01, respectively). However, the low level of green work climate was positively moderate and significant (conditional effect = 0.45, SE = 0.10, *t* = 4.27, *p* < 0.01, 95% confidence interval of 0.24-0.67) **(**Table 6 and Fig. 3**).**

**Table 6:** Moderator effect of the green climate

Moderator variable	Moderator level	Effect	SE	t	*p*	95% CI (LL)	95% CI (UL)
Green work climate	Low	0.45	0.10	4.27	0.0000	0.24	. 67
	Moderate	0.55	0.08	6.74	0.0000	0.39	0.71
	High	. 66	0.09	7.19	0.0000	0.48	0.85

## Discussion

The transformational leadership style of nursing plays a critical role in fostering a sustainable environment among nurses [26]. Nursing green transformational leaders use their behavior traits to inspire, motivate, and influence their nursing staff to engage in GB and foster their creativity in their workplace by establishing a favorable work climate [24, 58]. 

In this context, the present study aimed to investigate the effects of transformational leadership and a green work climate on nurses’ behavioral intentions, actual GB, and green creativity through the following: (1) investigating the relationships between nursing green transformational leadership, the green workplace climate, nurses’ green behavioral intentions, actual GB, and green creativity; (2) exploring the green climate as a mediating mechanism by which nursing green transformational leadership impacts nurses’ green behavioral intentions, actual GB, and green creativity; and (3) exploring the green climate as a moderating mechanism by which nurses’ green behavioral intentions affect nurses’ actual GB.

The current results revealed that nurses perceived nursing green transformational leadership, a green work climate, and their green behavioral intentions, actual GB, and green creativity in their workplace at acceptable levels. Similar results were reported in several studies in Egypt (2021 and 2024) [22, 23], Pakistan (2022) [59], and China (2023) [60].

This was in contrast to two Egyptian studies (2023 and 2024) [21, 61], which reported that nurses highly perceived leadership behavior displayed by immediate managers, a green climate, their GB, and creativity in their workplace. This contrast between the present study and other studies may be attributed to different types of leadership styles (ethical), nurse contexts, environmental attributes of the study setting and types of hospitals, in addition to sample sizes.

In the present study, nursing green transformational leadership had a statistically significant correlation and a significantly positive effect on the green work climate, nurses’ green behavioral intentions, actual GB, and creativity. The present work was similar to numerous industrial and tourism studies, which indicated that the transformational leadership style had positive effects on employee behavior and creativity by encouraging employees to act sustainably at work and be involved in innovative and environmentally friendly activities [27, 29, 35, 47, 59–61]. 

These results were also confirmed by an Egyptian study (2024) in healthcare organizations, which concluded that green transformational leaders influenced the GB and creativity of nurse managers [24]. The present findings are similar to those of other studies in China (2022) [15], Pakistan (2022)[59] and Saudi Arabia (2024) [1], which show that green transformational leaders can effectively engage their followers in actual GB and green creativity by providing them with enough support, resources, and appreciation in industrial settings.

The present findings can be explained by the fact that nurses in the study units may have perceived their managers as convenience role models who were able to set a clear environmental vision and apply good leadership behavior to inspire and motivate nurses to engage in GB and generate creative ideas and activities in their work. Leaders’ transformational behavior can help nurses engage in more GBs and see themselves as more creatively capable. Green transformational leaders influence employees through their green plans, visions, goals, beliefs, and ideas. The present study conforms to the findings of the Pakistani study (2023) [19] and the Egyptian study (2024) [24]. 

The results of the present study are supported by social exchange, social learning, and social influence theories, which state that green transformational leaders serving as “role models” can be perceived by employees [28, 33, 36–38, 42]. Leaders become role models for subordinates. They also build trust and caring relationships with their subordinates to stimulate their environmental awareness and carry out work that supports environmentally friendly behavior. Thus, this can make nurses work more eagerly and, in the end, can increase green creativity. Both leaders and employees can play a significant role in engaging in pro-environmental behaviors and creativity in the workplace [65]. The present findings are supported by the study of Farrukh et al. (2022) [47], who revealed that green transformational leaders have environmental role models that can influence employees’ environmental behavior and creativity.

The present findings were also confirmed by social influence and cognitive theories, which suggest that transformational leadership promotes intrinsic motivation in nurses [19, 35–37, 66]. Green transformational leaders can use their behaviors to influence employees to engage in more GB and motivate them to generate creative ideas. Thus, this style of leadership fosters employee creativity by increasing employee involvement in environmental protection through increased environmental awareness [29]. 

The present study also indicated that nursing green transformational leadership had a beneficial and significant effect on the green work climate. A similar result was reported in a study in South Africa (2023) [35]. The present findings align with those of a study in Iran (2020) [67] at twelve teaching hospitals in Tehran, which demonstrated that a nurse leadership style was found to be an important determinant and promoting factor for the organizational climate.

The present study also revealed that a green organizational climate played a mediating role between nursing green transformational leadership and nurses’ green behavioral intentions, actual GB, and creativity. Both nurses’ actual GB and creativity could be increased and improved if their leaders displayed nursing green transformational leadership in a favorable green work climate. Transformational leadership fosters a positive and supportive work environment where nurses feel valued and motivated. This enhances nurses’ overall behavior and creativity [5, 24, 30, 60, 66]. 

Additionally, these findings conform to the componential theory of creativity and social exchange theories, which suggests that green leadership plays a definite role in shaping a green climate, which is a factor in stimulating employees’ behavior and creativity. [28, 33, 41, 42].

These results can be attributed to nurses usually tending to engage in GBs and actively responding to generate green creative ideas when managers reasonably build positive organizational climates. A good green climate can help nurses recognize green healthcare activities, help them increase their awareness of environmental protection, and encourage them to exert extra effort during work or nonwork situations, thus increasing their willingness for GB and creativity [65]. 

Similar findings in different industrial and tourist organizations around the world have shown that a green transformational leadership style significantly impacts employees’ GB and creativity and that this relationship is mediated by the organizational climate [15, 20, 34, 35, 51, 68, 69]. 

The present study also indicated that a green climate was an essential contextual factor that positively affected GB. Nurses’ perceptions of a green work climate were found to be positively associated with their green behavioral intentions and actual GB. The impact of nurses’ green behavioral intentions on their actual GB was stronger for nurses who worked under a reasonable level of green work climate. Nurses’ green behavioral intentions promote their actual GB through the moderating effect of the green work climate. These results can be explained by nurses’ managers in the study units building a favorable green work climate and work environment characteristics that stimulate nurses’ green behavioral intentions to engage in actual GBs.

The results of the present work also support the theories of Rousseau (1985) [70] and Norton et al. (2014) [71], who suggested that the psychological climate was the best predictor of employee behavior because the psychological climate and employee behavior are individual-level constructs. The psychological climate is a key factor affecting employees’ positive behavioral outcomes. The present results are consistent with those of studies in China (2023) [72], Pakistan (2024) [48], and South Africa (2023) [35], which concluded that employees who have a more positive perception of a green work climate tend to be more involved in GBs.

The present study also revealed a strong positive correlation between nurses’ green behavioral intentions and their actual GB. The present study was supported by the theory of planned behavior, which states that individuals’ behavior is determined by their behavioral intentions [17]. This result was also consistent with a study in China (2021), which asserted that nurses’ green behavioral intention to engage in GB positively influenced their GB in three Chinese hospitals [16]. This finding aligns with the findings of Ansari (2024) [48], who reported that the work climate influences the link between intention and pre-environmental behavior in a significant and positive way.

### Theoretical implications

The current study provides five new key theoretical contributions to nursing management and environmental sustainability, specifically regarding the important role of green transformational leadership in shaping a green work climate and fostering nurses’ actual GB and creativity within healthcare settings.

First, the present study makes a novel contribution by being the first systematic investigation of the influence of green transformational leadership on nurses’ green behavioral intentions, actual GB, and creativity, significantly assisting nursing leaders in understanding sustainable healthcare management. Previous studies have focused mainly on investigating how green transformational leadership influences employees’ actual GB and creativity in the industrial and tourism fields [34, 60]. To the best of our knowledge, the role of green transformational leaders has not received much attention from researchers in the healthcare sector.

Second, this study contributes to the literature on the green transformation leadership of nurses working in healthcare settings through the use of creativity componential, social exchange, social cognition, and social influence theories. The current study links green transformational leadership with these theories to explore how green transformational leadership impacts nurses’ green behavioral intentions, actual GB, and creativity through the role of leadership –nurse relationships and fostering a positive green work climate [37, 38, 41, 42]. 

Third, this study offers actionable insights for assisting nursing leaders in healthcare sectors in understanding the ability of green transformational leadership practices to influence nurses’ green behavioral intentions, actual GB, and creativity through testing the mediating effects of the green work climate. To our knowledge, this approach has not been studied in the extant literature. The present study emphasizes that this specific topic requires more attention from healthcare managers and policymakers to align managerial perceptions with environmental sustainability, establish green practices, and mitigate climate change risks in healthcare systems [16]. 

Fourth, this study adds new strength to the concept that intentions come from the theory of planned behavior. Individuals’ behavior is determined by their intentions [16, 17]. Intentions depend on the green work climate. Intentions serve as key predictors of actual behaviors within the framework of a sustainable setting [13, 48]. A green work environment involves a culture, policies, and practices related to a sustainable environment in which employees work and follow green practices. This will enhance employees’ intentions to participate actively in environmentally responsible behaviors [48]. According to planned behavior theory, it is clear that people who have environmental concerns perform their tasks in a way that protects them [73]. 

Fifth, this study provides new insights into how nurses’ green behavioral intentions are translated into actual GB by moderating the green work climate. The green work climate acts as a boundary condition in the relationship between intention and actual GB. If it is not supportive, it may create barriers for nurses in translating their intentions into actual GB.

A green work climate is an essential contextual factor that positively affects GB. Employees usually tend to engage in GBs consistent with the perception of organizational policies and their feelings about what type of behaviors are rewarded by an organization. Thus, employees’ perceptions of their organization’s green climate motivate them to engage in GB in the workplace [32, 74]. The present study was based on the model of Norton et al. (2014) [71], which stated that the work climate was a key factor affecting employees’ positive behavioral outcomes (job engagement, organizational citizenship behavior, and employee performance). Previous studies in the hotel industry concluded that an optimistic green work climate reinforced the association between intention and GB, leading to increased engagement in green activities [75,76].

The study offers a direction to managers and policymakers in healthcare sectors on how to create a positive green work climate that promotes actual GB to foster green practices in healthcare settings. However, little attention has been given to exploring the moderating influence of the green work climate on the relationship between green behavioral intentions and actual GB [13, 48]. 

### Implications for nursing management

Green healthcare activities require leaders who improve their leadership style, are eager to set a clear environment vision, create a favorable green work climate, inspire nurses to engage in actual GB, and foster their green creativity. Therefore, green transformational leadership is essential for encouraging nurses to act sustainably at work, preserving the organizational environment and effectively achieving green healthcare activities.

In this respect, the present study draws upon certain green activity strategies that can be considered in healthcare organizations, such as setting clear workplace policies and procedures to guide nurses in green healthcare activities and establishing monitoring and evaluation mechanisms to reduce environmental hazards and preserve the environment.

These strategies will also include providing green transformational leadership training programs and seminars for all nursing managerial levels in different healthcare facilities to promote a green work climate that is beneficial for nurses, patients and organizations’ outcomes. These programs confirm the nursing-green transformational leadership role and the extent to which hospital and nursing staff could benefit from such green transformational leadership. It is crucial to encourage nursing managers to attend such programs and, more importantly, to support them by providing essential resources for implementing these programs.

Implementing such programs in the actual work situation can improve nurse managers’ GB through three main approaches: (1) providing eco-friendly knowledge about healthcare green practices and policies to motivate nurse managers to establish a green climate in their healthcare settings and perform more required GBs in their workplace; (2) teaching nurse managers how to create a green work climate in their daily work, allowing them to create autonomous motivational states and thereby engage in more GB and foster green creativity; and (3) training nurse managers on spontaneous GBs in their workplace, such as turning off lights and electrical devices when not in use in healthcare settings; disposing waste in appropriately color-coded bins; and sorting patient linens according to dirtiness during patient care and printing double-sided papers. Spontaneous GB is very important when environmental policies and systems are not clearly defined in healthcare organizations.

Nurse managers can foster their nursing green transformational leadership role by inspiring nurses with a clear sustainability vision and lead by role models, creating a supportive green work climate, building trustful and caring relationships with nurses, creating cross-functional collaboration teams and green committees to encourage nurse participation in sustainability activities within healthcare systems, establishing a green culture that encourages the generation of creative and new ideas, providing a high degree of autonomy to generate and share green ideas among nurses and establishing a reward system for motivating nurses to engage in actual GBs.

Furthermore, they should display a personal commitment to green practices in their workplace. This will increase nurses’ motivation and willingness to participate actively in actual GB.

Healthcare organizations also play a definite role in promoting green activities among nurses. They should disseminate green policies, procedures, and practices in such a way that nurses take an interest and have a positive attitude toward them. They may not only make policies and spread them to nurses but also provide resources and incentives to those who are involved in such activities.

Additionally, healthcare organizations should provide support for environmental awareness campaigns and design other green interventions, which will enable nurses to identify the importance of environmental sustainability and enhance their actual GB in the workplace.

Among many other challenges in nursing education is the embedding of green leadership and green healthcare practices into nursing administration courses to foster GBs and green creativity among undergraduate nursing students and meet the imperatives of the health service context of green activities.

The role of green leadership must be emphasized in the curricula by using diverse educational strategies to prepare nursing students to apply their professional role in green healthcare practices and activities. Nursing students should also be provided with support and empowerment to continuously improve their green leadership competencies. This would encourage them to demonstrate green leadership competencies in their clinical areas.

## Conclusion

The present study highlighted that nursing green transformational leadership contributed to the prediction of nurses’ green behavioral intentions, actual GB, and creativity. This effective relationship was influenced by an interaction effect between nursing green transformational leadership and a green work climate, especially when green work climate perceptions were acceptable. The green work climate served as a mediator between nursing green transformational leadership, nurses’ green behavioral intentions, actual GB and green creativity.

The study results also revealed the role of a green work climate as a moderator between green behavioral intentions and actual GB among nurses, thus enhancing nurse managers to identify the vital role of the green work climate in such an important relationship.

### Limitations of the study and further studies

Our study is the first to offer new insights and highlight the importance of green transformational leadership and a work climate in building actual GB and creativity among nurses in the nursing profession. However, our study has a few limitations that must be recognized to provide a new direction for additional research.

First, our study employed a cross-sectional design. Although it effectively captures data at a specific period, it would be necessary to collect data from the same nurses over an extended period to avoid any differences in the study results.

The cross-sectional design also limits the cause-and-effect relationship. Therefore, additional research should consider longitudinal designs to enhance the reliability of findings, account for temporal variations and establish causal relationships between the study variables effectively. Alternative study designs, such as experimental or quasi-experimental designs, in future research, are necessary to improve the validation of causality and reduce potential biases of a cross-sectional design.

Second, the generalizability of the results may be also constrained by the specific sample and healthcare organizational context. The study focused on teaching hospitals within Alexandria, which may restrict the generalizability of the results to nurses in other healthcare settings. Therefore, we need to conduct additional research in other healthcare settings, such as the Ministry of Health, insurance, and military hospitals.

Third, the present study focused exclusively on nurses engaged in direct patient care. Consequently, the perceptions of green work climate, GB, and green creativity may be particularly salient to this group. Future research should also explore these variables among senior nurses and those in managerial or administrative positions to determine whether similar patterns emerge across different professional levels within the nursing hierarchy.

Fourth, the current study only used nurses’ self-report questionnaires and self-evaluations from a single data source. This may affect the accuracy of the data and may add the risk of common method bias, including personal bias and potential response biases. Therefore, further research that collects more objective data from different data sources, such as head nurses, nursing supervisors, and directors, is needed.

Fifth, although the present study focused on the coupling role of green transformational leadership and a green work climate, we did not account for the effects of organizational culture. Organizational culture can affect not only the awareness of nurses and nursing leaders but also the development of ideals of leadership style and nurses’ GB. Therefore, future studies can consider organizational culture as a contextual factor.

Sixth, the present study did not examine the impact of nurses’ motivational factors, attitudes, or values on nurses’ GB and creativity. These variables may exert a significant influence on environmentally responsible behaviors. Future investigations should incorporate such constructs to develop a more comprehensive model of GB among nurses.

Additionally, to build on the findings of this study, future research is encouraged to identify and examine green citizenship behavior and green work engagement, which may further contribute to the adoption and implementation of green practices in healthcare settings.

Investigating individual differences, such as self-commitment as mediating factors could also offer valuable insights into the variability in nurses’ GB and creativity in future research. Furthermore, more control variables, such as qualifications and gender, can be also considered in future studies.

## Data Availability

All generated and analyzed data in this research are included in this manuscript. The datasets used and/or analyzed during the current study are available from the corresponding author upon reasonable request.

## References

[1] Abourokbah S, Bajaba S, Yaqub MZ. Leading the green wave: how and when green transformational leadership cultivates employee green creativity. Acta Psychol. 2024;250:104503. 10.1016/j.actpsy.2024.104503. 10.1016/j.actpsy.2024.104503 39357415

[2] Faraz NA, Ahmed F, Xiong X. How firms leverage corporate environmental strategy to nurture green behavior: role of multilevel environmentally responsible leadership. Corp Soc Responsib Environ Manage. 2024;31(1):243–59.

[3] Chen YS, Chang CH. The determinants of green product development performance: green dynamic capabilities, green transformational leadership, and green creativity. J Bus Ethics. 2013;116:107–19.

[4] Al-Sawy MMR. Greening the workplace: the power of a positive climate and leader influence on employee behavior. J Bus Res. 2023;45(3):1–45.

[5] Robertson JL, Barling J. Contrasting the nature and effects of environmentally specific and general transformational leadership. Leadersh Organ Dev J. 2017;38:22–41.

[6] Mansoor A, Farrukh M, Lee JK, Jahan S. Stimulation of employees’ green creativity through green transformational leadership and management initiatives. Sustainability. 2021;13(14):7844. 10.3390/su13147844.

[7] Gustiah IP, Nurhayati M. The effect of green transformational leadership on green employee performance through green work engagement. Sch J Econ Bus Manag. 2022;7159–68. 10.36347/sjebm.2022.v09i07.002.

[8] Yanhong L, Rafiq M. Transformational leadership: navigating green behavior through the ethical environment and overall justice. Published Online May. 2024;30. 10.1177/09722629241242630.

[9] Ismael HI, Sleem WF, Abd el-Ghani AM. Relationship between transformational leadership style and job satisfaction among nurses. Mansoura Nurs J (MNJ). 2024;11(1):357–68.

[10] Lathabhavan R, Kaur S. Promoting green employee behavior from the lens of green transformational leadership. Leadersh Organ Dev J. 2023;44(8):994–1015.

[11] Liang X, Li B, Gong Q, Li S, Guo G. Organizational green environment and employee green behavior: A moderated mediation model. ICPDI 2022 - Int Conf Public Manage Digit Econ Internet Technol. 2022;1(978–989–758–620–0):280–4.

[12] Daud ST, Suhaime LI, Sehat NS, Jogeran J. Leadership style that influences employees’ green behavior: A literature review. Int J Acad Res Econ Manage Sci. 2023;12(3):211–25.

[13] Norton TA, Zacher H, Parker SL, Ashkanasy NM. Bridging the gap between green behavioral intentions and employee green behavior: the role of green psychological environment. J Organizational Behav. 2017;38(7):996–1015.

[14] Schneider B, Ehrhart MG, Macey WH. Organizational environment and culture. Ann Rev Psychol. 2013;64:361–88. 10.1146/annurev-psych-113011-143809. 22856467 10.1146/annurev-psych-113011-143809

[15] Maitlo Q, Wang X, Jingdong Y, Lashari IA, Faraz NA, Hajaro NH. Exploring green creativity: the effects of green transformational leadership, green innovation environment, and green autonomy. Front Psychol. 2022;19(2):4106–29. 10.3389/fpsyg.2022.686373. 10.3389/fpsyg.2022.686373 PMC8964453 35369243

[16] Li M, Gong Z, Gilal FG, Van Swol LM, Xu J, Li F. The moderating role of ethical leadership on nurses’ green behavior intentions and real green behavior. Biomed Res Int. 2021:6628016:1–7. 10.1155/2021/6628016 10.1155/2021/6628016 PMC8068523 33954186

[17] Ajzen. The theory of planned behavior. Organ Behav Hum Decis Process. 1991;50(2):179–211.

[18] Poperwi L. The effect of green transformational leadership on green performance: A systematic review. In: Mhlanga D, Dzingirai M, editors. Fostering Long-Term sustainable development in africa: overcoming poverty, inequality, and unemployment. Switzerland: Springer Nature; 2024.

[19] Zainab Ya, Sajjad Hussain. Green transformational leadership: bridging the gap between green HRM practices and environmental performance through green psychological climate. Sustainable Futures. 2023;6:100140. 10.1016/j.sftr.2023.100140.

[20] Rehman FU, Zeb A. Investigating the nexus between authentic leadership, employees’ green creativity, and psychological environment: evidence from emerging economy. Environ Sci Pollut Res. 2023;30:107746–58. 10.1007/s11356-023-29928-1. 10.1007/s11356-023-29928-1 PMC10611599 37740164

[21] Ali OAA. Ethical leadership and its relationship with nursing staff green behavior and percieved green environment for environmental sustainability. Master degree in nursing administration, Faculty of nursing, Alexandria university, 2024.

[22] Essawy DRM, Gaber MA, Elaraby AE. Effect of ethical leadership on nurses’ green behavior at Zagazig university hospitals. Afr J Bio Sc. 2024;6(2):1932–44.

[23] Sorour MS, khairy HA, Elkholy SM. Relationship between servant leadership and its’ role on staff nurses’ creativity and sustainable development behavior. Assiut Sci Nurs J. 2021;9(24):87–101.

[24] Saleh MS, Elsabahy HE, Abdel-Sattar SA, Abd-Elhamid ZN, Al Thobaity A, Aly SMM, Mohamed Shokry WM. Fostering green transformational leadership: the influence of green educational intervention on nurse managers’ green behavior and creativity. Moustafa Saleh et al. BMC Nurs. 2024;23:393. 10.1186/s12912-024-01991-0. 38849843 10.1186/s12912-024-01991-0 PMC11157831

[25] Suliman MA, Abdou AH, Ibrahim MF, Al-KhaldyDA, Anas AM, Alrefae WM, Salama W. Impact of green transformational leadership on employees’ environmental performance in the hotel industry context: does green work engagement matter? Sustainability. 2023;15(3):2690. 10.3390/su15032690.

[26] Huang LZ, Guo B, Deng, Wang B. Unlocking the relationship between environmentally specific transformational leadership and employees’ green behavior: a cultural self-representation perspective. J Clean Prod. 2023;382:134–85.

[27] Du Y, Yan M. Green transformational leadership and employees’ taking charge behavior: the mediating role of personal initiative and the moderating role of green organizational identity. Int J Environ Res Public Health. 2022;19(4172). 10.3390/ijerph1907417. 10.3390/ijerph19074172 PMC8998811 35409857

[28] Liu X, Yu X. Green transformational leadership and employee organizational citizenship behavior for the environment in the manufacturing industry: A social information processing perspective. Front Psychol. 2023;13:1097655. 10.3389/fpsyg.2022.1097655. 36743625 10.3389/fpsyg.2022.1097655 PMC9891139

[29] Ding H, Su W, Hahn J. How green transformational leadership affects employee individual green Performance—A multilevel moderated mediation model. Behav Sci. 2023;13(887). 10.3390/bs13110887. 10.3390/bs13110887 PMC10669820 37998634

[30] Hameed Z, Naeem RM, Hassan M, Naeem M, Nazim M, Maqbool A. How GHRM is related to green creativity? A moderated mediation model of green transformational leadership and green perceived organizational support. Int J Manpow. 2022;43:595–613.

[31] Elshaer IA, Abdelrahman MA, Azazz AM, Alrawad M, Fayyad S. Environmental transformational leadership and green innovation in the hotel industry: two moderated mediation analyses. Int J Environ Res Public Health. 2022;19(16800). 10.3390/ijerph192416800. 10.3390/ijerph192416800 PMC9779271 36554691

[32] Saleem M, Qadeer F, Mahmood F, Ariza-Montes A, Han H. Ethical Leadership and Employee Green Behavior: A Multilevel Moderated Mediation Analysis. Sustainability Journal. 2020; 12 (8):3314. 10.3390/su12083314

[33] Amabile, T. M. In: Staw BM, Cummings LL. Research in organizational behavior. Stamford: JAI Press, 1988. pp. 123–167.

[34] Zakaullah, Raza SA, Saleem M, Muhammad A, Bilal M, Mahmood F. Green transformational leadership and employee’s green creative behavior: A moderated mediation analysis. Int J Contemp Bus Literature. 2024;4(1):21–9. 10.70890/IJCBL.2024.4103.

[35] Fatoki O. Green transformational leadership and employee pro-environmental behavior: The role of green thinking and green psychological climate. International Journal of Management and Sustainability. 2023;12(1):13-25.10.1016/j.heliyon.2024.e30096

[36] Xiao X, Tao X, Chen P, Kee DMH. Leading with purpose: Unraveling the impact of responsible leadership on employee green behavior in the workplace. Heliyon. 2024 10.1016/j.heliyon.2024.e30096 PMC11068640 38707323

[37] Bandura A. Social foundations of thought and action: A social cognitive theory. Englewood Cliffs, NJ: Prentice Hall; 1986.

[38] FriedkinNEA structural theory of social influence. Am J Sociol. 1998;45:162.

[39] Zafar H, Malik A, Gugnani R, Agarwal R, Nijjer S. Green thumbs at work: boosting employee eco-participation through ecocentric leadership, green crafting, and green human resource management. J Clean Prod, 2023; 432: 139718. doi:10.1016/j.jclepro.2023.139718.

[40] Zafar1 H, Suseno Y. Human resource management practices, green psychological climate, and organizational pride on employees’ voluntary Pro-Environmental behavior. Organ Environ. 2024;37(4):581–609.

[41] Amabile T. Componential theory of creativity. Boston, MA: Harvard Business School; 2011. pp. 538–59.

[42] Cropanzano R, Mitchell MS. Social exchange theory: an interdisciplinary review. J Manag.2005; 31(6), 874–900. doi:10.1177/ 0149206305279602.

[43] Afridi SA, Shahjehan A, Zaheer S, Khan W, Gohar A. Bridging generative leadership and green creativity: unpacking the role of psychological green climate and green commitment in the hospitality industry. SAGE Open.2023; 13(3). doi.:10.1177/21582440231185759.

[44] Flagstad I, Johnsen S. RydstedtLThe process of Establishing a green climate: Face-To-Face interaction between leaders and employees in the microsystem. J Values-Based Leadersh. 2021;14(1). 10.22543/0733.141.1343.

[45] O˘gretmeno˘glu M, Akova O, G¨oktepe S. The mediating effects of green organizational citizenship on the relationship between green transformational leadership and green creativity: evidence from hotels. J Hospitality Tourism Insights.2022; 5(4), 734–51. doi.:10.1108/JHTI-07-2021-0166.

[46] Srivastava S, Pathak D, Soni S, Dixit A. Does green transformational leadership reinforce green creativity? The mediating roles of green organizational culture and green mindfulness. J Organizational Change Manage. 2024. 10.1108/JOCM-09-2023-0364.

[47] Farrukh MN, Ansari A, Raza Y, Wu H, Wang. Fostering employee’s pro- environmental behavior through green transformational leadership, green human resource management and environmental knowledge. Technol Forecast Soc Change 2022; 179: 121643.

[48] Ansari HUIH, Khan SN. Linking green transformational leadership and employee preenvironmental behavior: the role of intention and work environment. Sustainable Futures. 2024;8(100336):1–9.

[49] Zaeni NA, Semmaila AB. The effect of compensation and work environment on employee performance, 3, point of view research management. 2022: 161–73.

[50] Faraz NA, Ahmed FZ, Xiong. How firms leverage corporate environmental strategy to nurture green behavior: role of multilevel environmentally responsible leadership. Corp Soc Responsib Environ Manage. 2024;31(1):243–59.

[51] Singh SCT. Green transformational leadership and pro-environmental behavior: unraveling the underlying mechanism in the hotel industry context. Int J Organizational Anal. 2024;32(2):255–71.

[52] Srivastava SD, Dixit SAP. Does green transformational leadership reinforce green creativity? The mediating roles of green organizational culture and green mindfulness. J Organizational Change Manage. 2024;37(3):619–40.

[53] Cochran WG. (1963): Sampling Techniques. (2nd ed.). New York: John Wiley & Sons. 1993; 7(3):.203-13.

[54] Norton TA, Zacher H, Ashkanasy NAO. The importance of pro-environmental organizational environment for employee green behavior. Industrial Organizational Psychology: Perspect Sci Pract. 2012;5:497–500.

[55] Bissing-Olson MJ, Iyer A, Fielding S, Zacher H. Relationships between daily affect and pro-environmental behavior at work: the moderating role of pro-environmental attitude. J Organizational Behav. 2013;34:156–75.

[56] Robertson JL, Barling J. Greening organizations through leaders’ influence on employees’ pro-environmental behaviors. J Organ Behav. 2013;34:176–94. 10.1002/job.1820.

[57] Ozolins SU, Hale X, Hyatt CH, Schoheld P. Translation and back-translation methodology in health. research–a critique. Expert Rev Pharmacoeconomics Outcomes Res. 2020;20(1):69–77. 10.1080/14737167.2020.1734453 32089017

[58] Ibrahim IA, El-Monshed AH, Gamal El-Sehrawy M, Elamir MG, Abdelrahim H. SM. Enhancing Nurses’ Well-Being: Exploring the Relationship between Transformational Leadership, Organizational Justice, and Quality of Nursing Work Life. Journal of Nursing Management. 2023 (2337975):1- 11. 10.1155/2023/2337975 10.1155/2023/2337975 PMC11919001 40225606

[59] Anwar I. Greening of Organizations; a theoretical framework linking Employees Perception about Green environment on Employees Green Behavior. Master thesis of management sciences capital, University of Science and Technology, Islamabad,2017.

[60] Huang ZL, Deng GB, WangB. Unlocking the relationship between environmentally specific transformational leadership and employees’ green behavior: a cultural self-representation perspective. J Clean Prod. 2023;382:134–85.

[61] El shrief HA, Salem HM, Ali HA. Humble leadership and its relation to staff nurses’ job crafting and creativity. Assiut Sci Nurs J. 2023;11(04):226–39.

[62] Alyahya M, Aliedan M, Agag G, Abdelmoety ZH. The antecedents of hotels’ green creativity: the role of green HRM, environmentally specific servant leadership, and psychological green climate. Sustainability. 2023;15(3):2629. 10.3390/su15032629.

[63] Sidney MT, Wang N, Nazir M, Ferasso M, Saeed A. Continuous effects of green transformational leadership and green employee creativity: a moderating and mediating prospective. Front Psychol. 2022;13:840019. 10.3389/fpsyg.2022.840019. 35645899 10.3389/fpsyg.2022.840019 PMC9139270

[64] Mittal S, Dhar RL. Effect of green transformational leadership on green creativity: a study of tourist hotels.tourism management. 2016; 57: 118–27.

[65] Saleem M, Qadeer F, Mahmood F, Ariza-Montes A, Han H. Ethical leadership and employee green behavior:a multilevel moderated mediation analysis. Sustainability. 2020;12(3314). 10.3390/su12083314.

[66] El sawah EE, Elkholy SM. Prosocial leadership and organizational sustainability: moderating role of nurses’ green behavior. IEJNSR. 2024;4(2):80–96.

[67] Aloustani S, Atashzadeh-Shoorideh F, Zagheri-Tafreshi M, Nasiri M, Barkhordari-Sharifabad M, Skerrett V. Association between ethical leadership, ethical environment and organizational citizenship behavior from nurses’. BMC Nurs. 2020;15. 10.1186/s12912-020-0408-1. 10.1186/s12912-020-0408-1 PMC7057459 32158354

[68] Wahba SAM, Al Asrag ASA, Hassan AHA, Majeed AHA. Green dynamic capabilities and green creativity in hospitality and tourism industry: the moderating role of green transformational leadership. JAAUTH. 2024;26(1):71–88.

[69] Zaid WMA, Yaqub MZ. The prolificacy of green transformational leadership in shaping employee green behavior during times of crises in small and medium enterprises: a moderated mediation model. Front Psychol.2024; 15:1258990. doi: 10.3389/fpsyg.2024.1258990. 38464624 10.3389/fpsyg.2024.1258990 PMC10920347

[70] Rousseau DM. Issues of level in organizational research: multilevel and cross-level perspectives. Res Organizational Behav. 1985;7(1):1–37.

[71] Norton TA, Hannes Zacher H, Ashkanasy NM. Organisational sustainability policies and employee green behavior: the mediating role of work climate perceptions. J Environ Psychol. 2014;38:49–54.

[72] Li ZH, Xing LY. The impact of green culture on employees’ green behavior: the mediation role of environmental awareness. Corp Soc Responsib Environ Manage. 2023;30(3):1325–35.

[73] Pai CJ, LePage BA, Ng E, Fang WT. Using the theory of planned behavior to examine the environmental behavior of roadrunners in Taiwan. Discover Sustain. 2024;5:535. 10.1007/s43621-024-00731-3.

[74] Moore C, Mayer DM, Chiang FF, Crossley C, Karlesky MJ, Birtch TA. Leaders matter morally: the role of ethical leadership in shaping employee moral cognition and misconduct. J Appl Psychol. 2019;104:123. 30221953 10.1037/apl0000341

[75] Sachdeva C, Singh T. Green transformational leadership and pro-environmental behavior: unraveling the underlying mechanism in the hotel industry context. Int J Organizational Anal. 2024;32(2):255–71.

[76] Rubel MRB, Kee DMH, Rimi NN. Green human resource management and supervisor pro-environmental behavior: the role of green work climate perceptions. J Clean Prod. 2021;313:127669.

